# RETRACTION: Effect of Continuous Nursing on Wound Infection and Quality of Life in Patients With Cancer‐Related Stoma: A Meta‐Analysis

**DOI:** 10.1111/iwj.70304

**Published:** 2025-03-06

## Abstract

**RETRACTION:** WeiS. , LiN. , LiX. , and QiM. , “Effect of Continuous Nursing on Wound Infection and Quality of Life in Patients with Cancer‐Related Stoma: A Meta‐Analysis,” International Wound Journal 20, no. 10 (2023): 3974–3980, 10.1111/iwj.14285.37376826 PMC10681417

The above article, published online on 27 June 2023, in Wiley Online Library (http://onlinelibrary.wiley.com/), has been retracted by agreement between the journal Editor in Chief, Professor Keith Harding; and John Wiley & Sons Ltd. Following an investigation by the publisher, all parties have concluded that this article was accepted solely on the basis of a compromised peer review process. The editors have therefore decided to retract the article. The authors did not respond to our notice regarding the retraction.

**RETRACTION:** S. Wei , N. Li , X. Li , and M. Qi , “Effect of Continuous Nursing on Wound Infection and Quality of Life in Patients with Cancer‐Related Stoma: A Meta‐Analysis,” International Wound Journal 20, no. 10 (2023): 3974–3980, 10.1111/iwj.14285.37376826 PMC10681417

The above article, published online on 27 June 2023, in Wiley Online Library (http://onlinelibrary.wiley.com/), has been retracted by agreement between the journal Editor in Chief, Professor Keith Harding; and John Wiley & Sons Ltd. Following an investigation by the publisher, all parties have concluded that this article was accepted solely on the basis of a compromised peer review process. The editors have therefore decided to retract the article. The authors did not respond to our notice regarding the retraction.

